# Epidemiologic Survey on *Toxoplasma gondii* and *Trichinella pseudospiralis* Infection in Corvids from Central Italy

**DOI:** 10.3390/pathogens9050336

**Published:** 2020-04-30

**Authors:** Francesca Mancianti, Giuliana Terracciano, Camilla Sorichetti, Giuseppe Vecchio, Daniele Scarselli, Stefania Perrucci

**Affiliations:** 1 Dipartimento di Scienze Veterinarie, Università di Pisa, Viale delle Piagge n. 2, 56124 Pisa, Italy; francesca.mancianti@unipi.it (F.M.); camilla.sorichetti@virgilio.it (C.S.); 2 Istituto Zooprofilattico Sperimentale delle Regioni Lazio e Toscana-Sezione di Pisa, Strada Statale Dell’Abetone e del Brennero, n. 4, 56123 Pisa, Italy; giuliana.terracciano@izslt.it; 3 Studio Agrofauna, Via dell’Artigianato n. 53/55, 57121 Livorno, Italy; vecchio@agrofauna.it (G.V.); info@agrofauna.it (D.S.)

**Keywords:** *Toxoplasma gondii*, *Trichinella pseudospiralis*, central Italy, magpie (*Pica pica*), hooded crow (*Corvus cornix*)

## Abstract

Free-ranging corvids—678 magpies (*Pica pica*) and 120 hooded crows (*Corvus cornix*) from nine protected areas of the Pisa province (central Italy)—were examined for *Toxoplasma gondii* and *Trichinella pseudospiralis*. The intracardiac blood clots from 651 magpies and 120 hooded crows were serologically examined for *T. gondii*. The DNA extracted from the hearts of seropositive birds was then used to perform a nested PCR for the amplification of the *T. gondii* B1 gene and for genotyping for SAG genetic markers. Breast muscle samples from 678 magpies and 91 hooded crows were tested by an artificial digestion method for *Trichinella*. Data were statistically analyzed. Forty-five (5.8%—41 magpies and four hooded crows) out of the 771 examined animals scored seropositive for *T. gondii,* with titers ranging from 1:25 to 1:100. *T. gondii* DNA was detected in 15 of the 45 positive birds and *T. gondii* genotypes II and III were identified. No positivity for *T. pseudospiralis* was found. No significant differences between the two species of corvids and among the different areas of origin were observed for seropositivity to *T. gondii*. This is the first extensive study on both *T. gondii* and *T. pseudospiralis* in magpies and hooded crows, as well as the first detection of *T. gondii* SAG genotypes in magpies.

## 1. Introduction

Magpies (*Pica pica*) and hooded crows (*Corvus cornix*) are sedentary birds with a scavenger behavior, feeding on carcasses, arthropods, vegetables, small prey and food waste [1]. Due to their feeding habits, these avian species may become infected by zoonotic parasites transmitted by the ingestion of oocysts and/or tissue infective stages, thus potentially contributing to the spread of these parasites and an increased risk of human infection [2,3].

Among zoonotic parasites, *Toxoplasma gondii* is an Apicomplexan species with felids as both definitive and intermediate hosts, whilst warm-blooded vertebrates, including birds, are intermediate hosts. Infection takes place by the ingestion of tissue cysts or of mature oocysts, shed by final hosts and sporulated in the environment. *T. gondii* has a cosmopolitan distribution and represents one of the most common human parasites [4,5]. In previous studies performed by molecular and/or serological analysis, different avian species have been found to be infected by *T. gondii* worldwide [6,7,8,9,10,11], with high prevalence values often observed in raptors and scavenger birds [1,7,12,13,14,15,16,17]. Nevertheless, to the best of our knowledge, data about *T. gondii* infections in corvids from European countries are scarce, being referred to in only two studies carried out in Spain [6,18].

*Trichinella* is a nematode genus whose adults live in the small intestine, while the infective larvae localize in the striated muscle of the same individual. Infection occurs following the ingestion of muscle larvae of infected hosts [19]. The genus *Trichinella* is a complex of at least 13 genotypes and can be divided into two clades: a clade consisting of “encapsulated” species/genotypes (including the species *Trichinella spiralis*, *Trichinella britovi*, *Trichinella nativa*, *Trichinella murrelli*, *Trichinella nelsoni*, *Trichinella patagoniensis*, *Trichinella chanchalensis* and the *Trichinella* genotypes T6, T8 and T9) and a “non-encapsulated” clade consisting of three species (*Trichinella papuae*, *Trichinella zimbabwensis*, and *Trichinella pseudospiralis*) [20,21].

All species of this genus can infect mammals, including humans, while only a few of them can infect birds or reptiles [20]. *T. pseudospiralis* is the only species capable of infecting both mammals and birds. It has a broad distribution area in Europe [22], and it is present in Italy, where it has been reported in wild mammals, humans and birds [23]. A role of birds in the spreading and maintenance of this species in nature has been suggested [23]. *T. pseudospiralis*-infected corvids could be eaten by other animals, such as rodents, which in turn can be consumed by wild boars or foxes. In this way, infected corvids may contribute to increasing the risk of human infections.

*T. pseudospiralis* infection has never been reported in European corvids, but it has been found in two specimens of *Corvus frugilegus* in Kazakhstan, Asia [2,23,24].

The present survey aimed to assess the occurrence of anti-*T. gondii* antibodies and of *T. gondii* DNA, perform a molecular genotyping, and assess the occurrence of *T. pseudospiralis* larvae in muscles, in magpies and in hooded crows living in faunistic areas of central Italy.

## 2. Results

Forty-five (41 magpies and 4 hooded crows) out of the 771 examined animals (5.8%) were found positive for anti-*T. gondii* antibodies, with titers ranging from 1:25 to 1:100. In detail, 3.3% (4/120) of the hooded crows and 6.3% (41/651) of the magpies scored positive. *T. gondii* DNA was detected in 15 out of the 45 positive specimens (2.3%). DNA was only successfully amplified in samples collected from magpies. The genotyping yielded eight genotypes II and seven genotypes III.

No statistically significant difference for seropositivity to *T. gondii* between the two species of corvids, neither among the different areas of origin, was found.

All birds examined for *T. pseudospiralis* were found negative.

## 3. Discussion

Hooded crows (*C. cornix*) and magpies (*P. pica*) are considered the most common corvid species in central Italy [25]. These animals are very versatile and opportunistic in finding food sources, and the feeding behavior of magpies is very similar to that of hooded crows, except for a few slight differences. In fact, feeding on a wide range of animals and vegetable foods, both animals are omnivorous [25]. Moreover, frequently feeding on carrions and food waste, they are both considered “scavenger” birds [1,25]. However, both these bird species may also feed on animals they hunt [26,27,28,29]. This feeding behavior has allowed a remarkable numerical expansion of populations of both these birds in Italy [30]. As a consequence, hooded crows and magpies are considered problematic species in Italy and other countries because of the damage they may cause to agricultural crops and because of the predation of passerine and galliform nests of hunting interest [25,31]. The feeding habits of these avian species slightly differ, with magpies being more able to consume greater quantities of insects than hooded crows. In addition, differently from hooded crows, magpies do not show differences in feeding habits according to the season [25]. Hooded crows, in fact, prefer feeding mainly on plants in the autumn–winter season, while in spring–summer their diet is mainly based on hunted animals or the remains of dead animals [25]. Finally, although magpies have a nesting attitude near human buildings or streets, hooded crows avoid nesting on trees near human settlements [29]. However, all the animals examined in this study lived in protected areas for faunistic restoration in the Province of Pisa, where human activities and settlements are strictly limited.

Toxoplasmosis is the most diffused parasitic zoonosis and a significant cause of health problems in humans worldwide [4,32,33]. Positive corvids are considered a potential source of *T. gondii* infection for other wild and domestic animals, including cats [32], thereby contributing to the transmission and the spread of the disease, and to an increased risk of human infection [33].

To the best of our knowledge, this is the first survey on *T. gondii* in corvids from Italy and the only European extensive study assessing *T. gondii* infection in these avian species, carried out by both serology and PCR.

The One Health approach recently suggested for this zoonotic infection recommends the monitoring of *T. gondii* prevalence by integrating human, domestic animals and wildlife data in the same area [4]. In northwest Tuscany, the seroprevalence of *T. gondii* infection in human populations has been investigated in a large sample of persons, resulting in an overall prevalence of 21.4% and, more specifically, of 19.4% in women of reproductive age [34,35]. Moreover, *T. gondii* infection has been previously reported with variable prevalence values in other wild animal species living in the same protected areas in the Province of Pisa, such as red foxes (53.4%), waterfowls (8.7%) and hares (0.9%) [10,36,37].

Despite corvids being included among potential sentinel animals for assessing the spread of *T. gondii* [1,6,25], the examined corvid population showed a low occurrence of toxoplasmic infection.

Similar studies in Europe, made on a smaller number of corvid specimens, have shown high seroprevalence rates (80.5%) in *Corvus corax* from Spain [18], while five out of 33 magpies were found positive for PCR [6]. Several crows (*Corvus* spp.) from Israel were found to be infected, with an overall seropositivity of 42.6% and the identification of the *T. gondii* genotype II from a seropositive animal [1]. Lastly, in a recent paper, a PCR prevalence of 16.36% was registered in nine out of 55 brain samples of hooded crows from public parks in Tehran, identifying the genotype III [38].

Along with corvid feeding behavior and sedentary habit, the main risk factors associated with high *T. gondii* seroprevalence in these birds include the age, the population density, the season and the density of human and feline populations in the area [1,8,32,33,39]. The low prevalence rates found in corvids examined in this study may be linked mainly to this latter factor, as all the carcasses examined here came from protected areas for faunistic restoration, where human activities and the presence of *T. gondii* final hosts are strictly limited.

In a previous investigation, the use of both heart and brain tissues was proven to enhance the sensitivity of testing for *T. gondii* DNA [11]. Therefore, although heart tissue is considered a more sensitive tissue in respect to brain tissue in chickens [5,7,11,40,41], the PCR prevalence rates recovered in the present study are probably underestimated, because only cardiac samples could be examined.

The identification of both SAG genotypes II and III appears in agreement with previous studies on similar avian species [1,38].

Although a role of birds in the spread of *T. pseudospiralis* has been suggested [23], a lack of parasitological evidence for *T. pseudospiralis* infection was observed in the examined corvids. These results agree with the findings of previous studies performed in several European countries, including Italy, Slovakia and Romania, which reported a negative result for all the examined birds of prey and corvids, magpies and crows included [42,43,44,45].

In Europe, *T. pseudospiralis* was found in different species of wild animals, such as birds of prey, wild boars, raccoons and red foxes, and was also reported in domestic pigs and rats [2,20,45,46,47,48]. Nevertheless, it is less common than encapsulated species and more common in mammals than in birds [20,23,24,48].

Although these latter findings can be related to the limited number of studies conducted on birds compared to studies concerning mammals [38,41], a lower risk of infection in birds rather than in mammals has also been suggested [20,23,38]. Moreover, according to Pozio [23], the role of birds in the epidemiology of *T. pseudospiralis* still needs to be elucidated. As mentioned, among corvids, *T. pseudospiralis* had previously been reported in only two specimens of *Corvus frugilegus* out of 732 examined corvids in Kazakhstan, Asia [2].

In Italy, *T. pseudospiralis* has been recently identified as the species responsible for some episodes of human infections, probably caused by the consumption of infected wild boar meat [http://www.ispalim.unina.it/Trichinella%20Pozio.pdf]. In birds from Italy, *T. pseudospiralis* has been reported only in nocturnal raptors in central Italy [49]. Despite the very low prevalence (1%), this finding may suggest that *T. pseudospiralis* could be endemic in this geographical area [49]. In addition, *T. pseudospiralis* has recently been reported in red foxes and wild boars in central Italy, and in some neighboring regions of northern Italy, such as Emilia Romagna and Liguria ([45], Deni personal communication).

## 4. Materials and Methods

A total of 798 wild corvids—678 magpies (*P. pica*) and 120 hooded crows (*C. cornix*)—of both sexes, provided by rangers of the Province of Pisa during the period December 2012–July 2013 and hunted in nine different areas for faunistic restoration located in eight different municipalities of the Province of Pisa (43°42’42”48 N, 10°24’52”92 E, Tuscany, central Italy) (Table 1), were examined. In these protected areas, human settlements and activities, as well as the presence of *T. gondii* final hosts, are strictly restricted.

Blood samples from 771 deceased animals (651 magpies and 120 crows) were collected from the heart or body cavities to determine the prevalence of anti-*Toxoplasma* antibodies. Serum samples were examined by modified agglutination test (MAT) using Toxoscreen DA^®^, (Biomérieux, Lyon, France), starting from a 1/25 dilution as reported by Molina Lopes et al. [18].

Portions of myocardial tissue (about 10 g) and blood samples from seropositive subjects were selected, homogenized and used to perform PCR analysis for *Toxoplasma* DNA using the QIAamp^®^ DNA minikit (Qiagen, Milan, Italy) in accordance with the manufacturer’s instructions.

PCR for the amplification of the B1 gene was performed as reported by Jones et al. [50]. The genotypes were characterized by using five genetic markers (SAG1, 3′-SAG2, 5′-SAG2, alt. SAG2, SAG3) as described by Su et al. [51], modified.

The artificial digestion technique was the elective method for the diagnosis of *Trichinella* spp. in infected animals [42], with the pectoral, head and neck muscles considered as the more sensitive samples in birds [23,24,42,49,52]. In this study, breast muscle samples (5 g each) from 678 magpies and 91 hooded crows were tested by artificial digestion following the method reported by the European Commission Regulation 2075/2005 [52] for the search of *T. pseudospiralis* larvae. After digestion, the larvae that were eventually recovered from the muscle were processed for molecular typing [20,21,22,23].

The differences in the prevalence of *T. gondii* in the two corvid species and in the nine different areas of the Pisa province examined in this study were statistically analyzed using the statistical package SPSS Advanced Statistics 13.0 (SPSS Inc., Chicago, IL, USA). The A χ2 test with the Yates correction was chosen as the reference test. Results were considered statistically significant when *p* < 0.05.

## 5. Conclusions

This is the first extensive study aiming to evaluate the occurrence of both *T. gondii* and *T. pseudospiralis* in magpies and hooded crows, as well as the first report on the detection of *T. gondii* SAG genotypes in magpies. Although the feeding habits of the examined avian species would predispose them to toxoplasmic infection, the prevalence rates found were quite low. Despite the documented presence of *T. pseudospiralis* in wild animals from Tuscany and some neighboring regions, especially in wild boars and foxes, the results obtained in this study seem to indicate that in the examined areas, corvids do not harbor *T. pseudospiralis*.

## Figures and Tables

**Table 1:** Faunistic areas and municipalities of origin of the 798 wild corvids—678 magpies (*Pica pica*) and 120 hooded crows (*Corvus cornix*)—of both sexes, examined for anti-*Toxoplasma* antibodies and *Toxoplasma gondii* DNA, as well as for larvae of *Trichinella pseudospiralis* in the muscle.

	Magpies		Hooded Crows	
	*Trichinella*	*Toxoplasma*	*Trichinella*	*Toxoplasma*
**Faunistic Area (Municipality)**	N. of birds	N. of birds	N. of birds	N. of birds
**Asciano** **(S. Giuliano Terme)**	27	28	15	15
**Bientina (Bientina)**	-	104	-	-
**Calcinaia (Calcinaia)**	52	55	20	22
**Casaglia (Montecatini Val di Cecina)**	68	68	8	7
**Casale Marittimo** **(Casale Marittimo)**	163	158	-	-
**Guardistallo (Guardistallo)**	99	50	-	-
**Navacchio (Cascina)**	71	69	25	25
**Querceto (Montecatini Val di Cecina)**	138	58	23	51
**Rio Arbiaia (Pomarance)**	60	61	-	-
**Total**	678	651	91	120
